# Linking Item Response Model Parameters

**DOI:** 10.1007/s11336-015-9469-6

**Published:** 2015-07-09

**Authors:** Wim J. van der Linden, Michelle D. Barrett

**Affiliations:** CTB/McGraw-Hill Education, 20 Ryan Ranch Road, Monterey, CA 93940 USA

**Keywords:** 3PL response model, item calibration, linking design, linking function, parameter identifiability

## Abstract

With a few exceptions, the problem of linking item response model parameters from different item calibrations has been conceptualized as an instance of the problem of test equating scores on different test forms. This paper argues, however, that the use of item response models does not require any *test score equating*. Instead, it involves the necessity of *parameter linking* due to a fundamental problem inherent in the formal nature of these models—their general lack of identifiability. More specifically, item response model parameters need to be linked to adjust for the different effects of the identifiability restrictions used in separate item calibrations. Our main theorems characterize the formal nature of these linking functions for monotone, continuous response models, derive their specific shapes for different parameterizations of the 3PL model, and show how to identify them from the parameter values of the common items or persons in different linking designs.

## Introduction

The literature on item response model parameter linking tends to conceptualize the problem of linking the parameters from different calibrations as a step in the process of test score equating. For instance, for the well-known dichotomous logistic response models, Kolen and Brennan (2004, p. 156) treat the linking problem as the second step in a three-step process consisting of (i) estimating the item parameters in the response model for a new test form, (ii) scaling the parameters back to a base scale using a linear transformation, and (iii) if number-correct scoring is used, number-correct scores on the new form are converted to number-correct scores on an old form and then to scale scores. References to the problem addressed in this paper as an equating problem are also found, for instance, in Dorans, Pommerich, and Holland (2007), Holland and Rubin (1982), and von Davier (2011).

The main model considered in this paper is the fixed-effects three-parameter logistic (3PL) response model, which explains the probability of a correct response $U_{pi}=1$ for a test taker *p* on item *i* with ability $\theta _{p}\in \mathbb {R}$ as

1 $$\begin{aligned} \Pr \{U_{pi}=1;\theta _{p}\}\equiv p(\theta _{p};a_{i},b_{i},c_{i})\equiv c_{i}+(1-c_{i})\Psi [a_{i}(\theta _{p}-b_{i})], \end{aligned}$$

with

2 $$\begin{aligned} \Psi [a_{i}(\theta _{p}-b_{i})]\equiv \frac{\exp [a_{i}(\theta _{p}-b_{i})]}{1+\exp [a_{i}(\theta _{p}-b_{i})]}. \end{aligned}$$

where $b_{i}\in \mathbb {R}$ and $a_{i}>0$ are parameters for the difficulty and discriminating power of item *i*, respectively, and $c_{i}\in (0,1]$ represents the height of the lower asymptote to the probability for the item. Let $\theta ^{*}$ and $\theta$ denote the abilities of arbitrary test takers on an old and new test form calibrated under this model. The linear transformation used in the second step above is

3 $$\begin{aligned} \theta ^{*}=u\theta +v, \end{aligned}$$

with parameters *u* and *v* to be derived from response data for the two forms.

The standard argument for the claim of linearity of the transformation in the literature relies on the notion of indeterminacy of the scale of the (estimated) $\theta$ scores (e.g., Kim, Harris, & Kolen, 2010, p. 264; Lord, 1980, sect. 3.5). More precisely, it points at an arbitrary zero and unit for these scores, which manifest themselves by the fact that we can always transform $\theta$ as in (3), provided the two remaining parameters in (2) are replaced by

4 $$\begin{aligned} a_{i}^{*}=a_{i}/u \end{aligned}$$

and

5 $$\begin{aligned} b_{i}^{*}=ub_{i}+v. \end{aligned}$$

for all *i*.

Popular methods to estimate the parameters *u* and *v* are the mean/sigma method (Macro, 1977), the mean/mean method (Loyd & Hoover, 1980), and the methods based on the entire response functions for the common items in the two test forms by Haebara (1980) and Stocking and Lord (1983). The first two methods are based on a choice from the following relationships between the parameter values in the two calibrations:

6 $$\begin{aligned} u= & {} \frac{\mu (a)}{\mu (a^{*})},\text { }\mu (a^{*})>0, \end{aligned}$$

7 $$\begin{aligned}= & {} \frac{\sigma (b^{*})}{\sigma (b)},\text { }\sigma (b)>0, \end{aligned}$$

8 $$\begin{aligned}= & {} \frac{\sigma (\theta ^{*})}{\sigma (\theta )},\sigma (\theta )>0. \end{aligned}$$

and

9 $$\begin{aligned} v= & {} \mu (b^{*})-u\mu (b) \end{aligned}$$

10 $$\begin{aligned}= & {} \mu (\theta ^{*})-u\mu (\theta ), \end{aligned}$$

with $\mu (\cdot )$ and $\sigma (\cdot )$ denoting means and standard deviations. These methods are applied substituting the means and/or variances of the parameter estimates for the common item and persons in the linking study in these expressions. The *BILOG* computer program (Mislevy & Bock, 1990) included a version of these methods with the arithmetic means of the *b* parameters in (9) but the geometric instead of the arithmetic means of the *a* parameters in (6); for a generalization of this log-mean/mean procedure, see Haberman (2009). The Stocking-Lord method finds estimates of *u* and *v* minimizing the squared difference between the sums of the response functions for common items in the two test forms. For the three-parameter logistic (3PL) model in (1), the criterion to be minimized is

11 $$\begin{aligned} \left[ \sum \limits _{i}p(\theta ;a_{i}^{*},b_{i}^{*},c_{i}^{*})-\sum _{i}p(\theta ;a_{i}/u,ub_{i}+v,c_{i})\right] ^{2}, \end{aligned}$$

for a selection of $\theta$ values and with estimates substituted for the item parameters. For further details on these methods, see Kolen and Brennan (2004, sects. 6.2–6.3).

From a practical point of view, this treatment of the linking of response model parameters as a step in test score equating seems to make sense. IRT parameter linking does have some history in the context of IRT observed-score equating (Lord, 1980). And the fact that the necessary data for the estimation of the linear transformation in (3) are collected using the same type of sampling designs as used in plain observed-score equating (equivalent-groups designs; anchor-item designs; etc.) seems to lend additional support to the treatment of IRT parameter in this context.

From a more theoretical perspective, however, objections to this point of view are possible. First of all, a characteristic feature of all item response models is the presence of separate parameters for the effects of the properties of the items and the test takers’ abilities on the response probabilities. A naïve observer may note that the item parameters already adjust the probabilities for the differences between the items in the test forms and wonder where the necessity of the additional linking does come from.

A more fundamental puzzle is the assumed linearity of the linking transformation. The argument of the logistic function in (2) is definitely nonlinear (products of $a_{i}$ with $\theta _{p}$ and $b_{i}$). So it would be wrong to use this aspect of the parameter structure to motivate the shape of the transformation. Further, although the notion of a measurement scale for an ability with an indeterminate zero and unit has a long tradition in the behavioral and social sciences, enforced by classical publications such as Stevens (1946), its use in the current context focuses our attention exclusively on the scale of the $\theta$ parameter that is measured. But the model in (1)–(2) has four parameters for each response probability. Why should we be interested in the $a_{i}$ and $b_{i}$ parameters only because of features of the scale of $\theta$? And how about the $c_{i}$ parameters? If their scale is determinate, how come the Haebara and Stocking-Lord methods, which are sensitive to estimation error in these parameters, have been claimed to outperform the mean/mean and mean/sigma methods (e.g., Baker & Al-Karni, 1991)? But if their scale is indeterminate, how could we ever motivate the popularity of the last two methods in the linking literature, which ignore the $c_{i}$ parameter completely?

Similar questions arise if we reparameterize the model. For example, for computational reasons (use of the Gibbs sampler), it has become convenient for Bayesian estimation to reparameterize the argument of the logistic function in (2) as $\alpha _{i}\vartheta _{p}+\beta _{i}$ (Albert, 1992). But does the scale of $\vartheta$ for this version of the model still have an indeterminate zero and unit? And is its linking transformation still linear?

The view of parameter linking in this paper is solely as a fundamental problem due to a formal feature of item response models—their general lack of identifiability. The notion of model identifiability seems akin to the one of indeterminacies of scales in Stevens’ (1946) classification of measurement scales. But, unlike Stevens’ classification, which just consists of a set of definitions of different levels for the scale of the parameter we try to measure and then lets us wonder how to establish the nature of the scale in a specific measurement situation, it implies a formal criterion that can be applied directly to the measurement model that is used. Loosely speaking, the criterion requires us to check if each possible distribution of the response data implies a unique set of values for *all* parameters in the model.

If a models lacks identifiability, the problem can be resolved by adjusting its numbers of equations and/or parameters, which in the current context of IRT parameter estimation with given numbers of item and person parameters leads to the necessity of extra restrictions on them. The well-known practice of setting the mean and standard deviation of the ability parameters in a maximum marginal likelihood (MML) calibration of the items equal to

12 $$\begin{aligned} \mu _{\theta }=0,\sigma _{\theta }=1 \end{aligned}$$

is an example of the use of such restrictions.

However, even if the problem of identifiability is resolved, a new problem arises. The general effect of the use of identifiability restrictions is different values for the parameters of the same items and test takers in different calibrations. Hence, these parameters can only be compared if we know the function that maps the set of their values for one calibration onto those for the other. Once all parameters are linked, the response model automatically adjusts for any relevant differences between items or test takers, and future estimates of any ability parameter for given item parameters (or reversely) are always directly comparable. Thus, linking functions are not necessary to correct for arbitrary units and zeroes of the $\theta$ parameters but, more generally, *to adjust for the different effects of the identifiability restrictions used in separate calibrations*.

Observe that the differential effect also arises if we use (12 ) for two separate calibrations. Using superscripts to index different groups of test takers, we then have

13 $$\begin{aligned} \mu _{\theta }^{(1)}=0,\quad \sigma _{\theta }^{(1)}=1 \end{aligned}$$

and

14 $$\begin{aligned} \mu _{\theta }^{(2)}=0,\quad \sigma _{\theta }^{(2)}=1. \end{aligned}$$

The prevalent practice of not indexing different groups of test takers in (12) may easily lead to the erroneous belief that these restrictions always have the same effect. However, as explained in more detail below, each of these two sets of restrictions yields a different intersection with the model equations and hence different identified values for all model parameters.

As just noted, linking functions are thus mathematical functions that map the set of values for the item and ability parameters in the response model for one calibration onto those for another that are necessary because of its lack of identifiability. The main theorems in this paper characterize these functions for the general class of monotone, continuous item response models, and derive their specific shapes for different parameterizations of the 3PL model with the fixed ability parameters in (1)–(2 ). In addition, they show how to identify the linking functions from the parameter values of common items or persons for different linking designs. As the current focus is only on the mathematical definition of linking functions, we treat all item and ability parameters as known and postpone the treatment of the statistical problem of estimating such functions. Before presenting the theorems, a few notions from the literature on model and parameter identifiability necessary to understand the nature of these functions are reviewed.

## Observational Equivalence and Identifiability

We restrict the review of the problem of identifiability to the class of models that serve as parametric probability functions for the distribution of (discrete) random variables. Except for an occasional definition (e.g., Casella & Berger, 2002, sect. 11.2), the problem does not have much of a history in textbooks on statistics. All families of distributions typically discussed in these texts have probability functions with standard parameters that are identifiable. But the problem does have an active history of research in econometrics, mainly because of its tradition of modeling these standard parameters as functions of quantities of substantive interest, as well as in generalized latent variable modeling. Classical papers in the econometric literature discussing parameter identifiability include Koopmans (1949), Fisher (1961; 1965), Rothenberg (1971), Richmond (1974), and Gabrielsen (1978). Bekker, Merckens, and Wansbeek (1994) offer an enlightening analysis of the problem of identifiability in structural equation modeling. For an introduction from the perspective of generalized latent variable modeling, see Skrondal and Rabe-Hesketh (2004, chap. 5).

The problem of identifiability does arise in IRT because of its similar attempt to explain standard parameters of response distributions as functions of item and person parameters. Relevant papers addressing the problem for a variety of response models include Bechger, Verhelst, et al. (2001), Bechger, Verstralen, et al. (2002), Fischer (2004), Maris (2002), Maris and Bechger (2004; 2009), Reiersøl (1950), Revuelta (2009), San Martín, Gonzáles, and Tuerlinckx (2009), San Martín, Jara, et al. (2011), Tsai (2000), and Volodin and Adams (2002). The problem of linking parameters estimated under different identifiability restrictions seems to be restricted mainly to item response theory, however. At least, these authors are not aware of any other field where parameters estimates are linked as frequently and routinely as in educational and psychological testing; the only exception known to them is Luijben’s (1991) treatment of the equivalence of two differently restricted versions of an unidentifiable structural equations model (also addressed by Bekker et al. 1994, chap. 7). For the treatment of parameter linking for a response-time model with item and person parameters from the perspective of model identifiability, see van der Linden (2010).

The following definitions, which can be found throughout the literature just referred to, are for a model of a random vector with a multidimensional parameter space:

### **Definition 1**

Two points in a parameter space are observationally equivalent if they imply the same joint distribution of the random vector.

### **Definition 2**

A parameter is identifiable if for any of its points there is no other point that is observationally equivalent.

More formally, let $\mathbf {x}$ denote the random vector that is considered and $f(\mathbf {x};{\varvec{\pi }})$ its probability function, which is assumed to have vector-valued parameter ${\varvec{\pi }}$. Then, ${\varvec{\pi }}$ is identifiable if for any pair ${\varvec{\pi }}_{0}\ne$ ${\varvec{\pi }}_{1}$, it holds that $f(\mathbf {x};{\varvec{\pi }}_{0})\ne f(\mathbf {x}; {\varvec{\pi }}_{1})$ for all $\mathbf {x}$. Observe that lack of identifiability may be due to some of the components of ${\varvec{\pi }}$ only. As $\mathbf {x}$ typically represents observed data, it is common to refer to a parameter as being “identifiable from the data.” The use of this phrase emphasizes the practical meaning of parameter identifiability: If different values of ${\varvec{\pi }}$ imply the same probability distribution, it becomes impossible to use observed data to distinguish between them, let alone infer a “true” value of ${\varvec{\pi }}$. Consequently, if ${\varvec{\pi }}$ is not identifiable, then for some of its values, the likelihood function $f({\varvec{\pi }} ;{{\mathbf x}})$ associated with the observations does not allow us to discriminate between them. Indeed, if a parameter lacks identifiability, it does not have a consistent estimator (Gabrielsen, 1978).

Definition 2 immediately suggests possible refinements of the criterion of identifiability, such as local identifiability of ${\varvec{\pi }}$ at ${\varvec{\pi }}_{0}$ (i.e., ${\varvec{\pi }}$ is identifiable in a neighborhood of ${\varvec{\pi }}_{0}$) or identifiability from a restricted set of values of *x*. But more important to our current goals is a discussion of a slightly generalized version of a theorem in Bartels (1985):

### **Theorem 1**

If ${\varvec{\pi }}$ and ${\varvec{\varphi }}$ are the parameters in alternate versions of a model of a given random variable that have a bijective relationship**,** then ${\varvec{\pi }}$ is identifiable if and only if ${\varvec{\varphi }}$ is.

The theorem is immediately obvious if we realize that, in the current context, parameters serve as quantities that index individual members of families of probability distributions. As the relation between ${\varvec{\pi }}$ and ${\varvec{\varphi }}$ is bijective (one-to-one and onto), their role as index is entirely exchangeable.

The theorem explains why we can reparameterize a model (replace its structure with one set of parameters by a structure with another set) without losing its identifiability, provided the two sets of parameters have a bijective relationship. An example is the slope-intercept parameterization $\alpha _{i}\vartheta _{p}+\beta _{i}$ of the logistic model referred to earlier. In order to give the response function this parameter structure, we have to substitute

15 $$\begin{aligned} \theta _{p}= & {} \theta (\vartheta _{p})=\vartheta _{p}; \nonumber \\ a_{i}= & {} a(\alpha _{i})=\alpha _{i}; \\ b_{i}= & {} b(\alpha _{i},\beta _{i})=-\beta _{i}/\alpha _{i}, \nonumber \end{aligned}$$

$\vartheta _{p},\beta _{i}\in \mathbb {R}$ and $\alpha _{i}>0$, into (2). The relation between the two alternative sets of parameters is invertible, and thus bijective.

A special instance of the function ${\varvec{\varphi }}=\varphi ({\varvec{\pi }} )$ in Theorem 1 is the vector function

16 $$\begin{aligned} {\varvec{\varphi }} =(\varphi _{1}(\pi _{1}),\ldots ,\varphi _{d}(\pi _{d})), \end{aligned}$$

with $\varphi _{1},\ldots ,\varphi _{d}$ being scalar-valued, bijective functions of the *d* different components of ${\varvec{\pi }}$. An example of this *componentwise* type of bijective function is the well-known reparameterization of the Rasch model,

17 $$\begin{aligned} p(\vartheta _{p};\beta _{i})\equiv \frac{\vartheta _{p}}{\vartheta _{p}+\beta _{i}}, \end{aligned}$$

$\vartheta _{p},\beta _{i}>0$, which, maintaining our current notation, follows from (2) with $a_{i}=1$ upon substitution of

18 $$\begin{aligned} \theta _{p}= & {} \theta (\vartheta _{p})=\ln \vartheta _{p}; \nonumber \\ b_{i}= & {} b(\beta _{i})=\ln \beta _{i},\text { }\vartheta _{p},\beta _{i}>0. \end{aligned}$$

The critical difference between the two types of reparameterization resides thus in the fact that *each* component in (18) is a bijective function of its counterpart as well, whereas the component for $$ b_{i}$$ in (15) is not.

Reparameterization is a useful tool if we have to prove identifiability of a model. It allows us to replace the set of model equations with the current parameters by an equivalent set for which the proof is simpler. We will use this trick to prove some of our later results. Also, the problem of parameter linking will appear to be one in which we have to derive and estimate functions as in (16).

As already noted, the typical solution to an identifiability problem for an item response model is the introduction of extra restrictions on its parameters. Their effect is a reduction of the parameter space to one that uniquely represents each possible member of the family of response distribution posited by the model. However, such restrictions generally have a differential impact on the parameter spaces in different calibrations, lead to different unique representations, and consequently leave us with a linking problem. Because our focus is mainly on this problem, we only highlight the nature of the identifiability problem for the 3PL model in (1)–(2), presenting a few cases in which different sets of parameters in the 3PL model in (1)–(2) clearly show lack of identifiability (including the commonly believed to be invariant $c_{i}$ parameters). It is not our intention to provide a solution for it. In fact, as follows from Theorem 3 below, in order to derive a linking function for monotone continuous response model, it is not necessary to know the identifiability restrictions actually used in the different calibrations at all; only their differential impact on the item and ability parameters values counts.

## 3PL Model

The distributions addressed by the 3PL model in (1) are for the dichotomous responses $U_{pi}=0,1$ by test takers $p=1,\ldots ,P$ on items $i=1,\ldots ,I$. The distributions are Bernoulli with probability functions

19 $$\begin{aligned} f(u_{pi};\pi _{pi})=\pi _{pi}^{u_{pi}}(1-\pi _{pi})^{1-u_{pi}},\text { } p=1,\ldots ,P;\text { }i=1,\ldots ,I, \end{aligned}$$

which have success parameters $\pi _{pi}\in [0,1]$ representing the probability of a correct response by each test taker on each item.

Each of these probability functions is identifiable because their only parameter, $\pi _{pi}$, is; for different values of this parameter (19) always yield different distributions. Observe that if these probability functions are reparameterized by substituting $\pi _{pi}=1-\eta _{pi}$, Theorem 1 guarantees that $\eta _{pi}$ is also identifiable. We will use this feature frequently.

Making the usual assumption of independence within and between test takers, the probability function of the joint distribution of a complete response matrix, $\mathbf {U}=(U_{pi})$, is the product of $P\times I$ of these Bernoulli distributions,

20 $$\begin{aligned} f(\mathbf {u};{\varvec{\pi }})=\prod _{p}\prod \limits _{i}\pi _{pi}^{u_{pi}}(1-\pi _{pi})^{1-u_{pi}} \end{aligned}$$

with parameter vector ${\varvec{\pi }}=(\pi _{11},\ldots ,\pi _{1I},\ldots ,\pi _{P1},\ldots ,\pi _{PI}).$ Clearly, as each of its components is identifiable, so is ${\varvec{\pi }}$.

The 3PL model specifies each $\pi _{pi}$ as a function of the parameters ($\theta _{p},a_{i},b_{i},c_{i}$) for the effects of the test taker’s ability and the properties of the item on it. Rather than a direct probability function for a response distribution, the model is thus a (second-level) mathematical model in the form of a system of $P\times I$ nonlinear equations

21 $$\begin{aligned} \pi _{pi}=c_{i}+(1-c_{i})\Psi [a_{i}(\theta _{p}-b_{i})],\text { } p=1,\ldots ,P;\text { }i=1,\ldots ,I, \end{aligned}$$

one for each of the success parameters.

### Lack of Identifiability

The following three cases illustrate the lack of identifiability of the 3PL model:

#### **Theorem 2**

The system of equations for the 3PL model in (21) is not identifiable in the following cases: (i) $c_{i}$ known for all *i*; (ii) $a_{i}=a\in \mathbb {R} ^{+}$, for all *i*; and (iii) $\theta _{p}=\theta \in \mathbb {R}$ for all *p*.

#### *Proof*

(i) This case basically amounts to the 2PL model in (2). The fact that different values of $$a_{i},$$ $$ b_{i},$$ and $$\theta _{p}$$ parameters do not need to imply different values for $$\pi _{pi}$$ follows from (3)–(5), and is well known. (ii) Lack of identifiability of the $$b_{i}$$, $$c_{i}$$, and $$\theta _{p}$$ parameters for this case was established by Maris (2002) and later re-analyzed in Maris and Bechger (2009). Without loss of generality, his proof sets $$a=1$$ and reformulates (3)–(5) as

22 $$\begin{aligned} \pi _{pi}=\frac{\exp (\theta _{p})+c_{i}\exp (b_{i})}{\exp (\theta _{p})+\exp (b_{i})}, \end{aligned}$$

which, upon substitution of $\theta _{p}=\ln \vartheta _{p}$, $b_{i}=\ln \beta _{i}$, and $c_{i}=\ln \delta _{i}/\beta _{i},$ leads to

23 $$\begin{aligned} \pi _{pi}=\frac{\vartheta _{p}+\delta _{i}}{\vartheta _{p}+\beta _{i}}. \end{aligned}$$

Adding a constant to $\vartheta _{p}$ for all *p* and subtracting the same constant from $\beta _{i}$ and $\delta _{i}$ for all *i* give different sets of values for these parameters with the same $\pi _{pi}$. (iii) Let $\pi _{pi}=1-\eta _{pi}$ and $c_{i}=1-\gamma _{i}$. Theorem 1 implies that $\pi _{pi}$ and $c_{i}$ are identifiable if and only if $\eta _{pi}$ and $\gamma _{i}$ are. From (1)–(2), using $\theta _{p}=\theta$ for all *p*,

24 $$\begin{aligned} \eta _{i}= & {} \frac{\gamma _{i}}{[1+\exp (a_{i}(\theta -b_{i}))]} \nonumber \\= & {} \frac{\gamma _{i}^{*}}{\kappa [1+\exp (a_{i}(\theta -b_{i}))]}, \end{aligned}$$

for all *i* and any $\kappa \in (0,\gamma _{i}]$, where $\gamma _{i}^{*}=\kappa \gamma _{i}$. In order to have $b_{i}$ absorb $\kappa$, we need to substitute $b_{i}^{*}$ for $b_{i}$, where $b_{i}^{*}$ is the solution of

25 $$\begin{aligned} 1+\exp (a_{i}(\theta -b_{i}^{*}))=\kappa [1+\exp (a_{i}(\theta -b_{i}))], \end{aligned}$$

which is

26 $$\begin{aligned} b_{i}^{*}=\theta -\ln (\kappa [1+\exp (a_{i}(\theta -b_{i}))]-1]/a_{i}. \end{aligned}$$

For the relation in (26) to hold, it is necessary that $\kappa [1+\exp (a_{i}(\theta -b_{i}))]-1>0$; or,

27 $$\begin{aligned} \kappa> & {} \frac{1}{1+\exp (a_{i}(\theta -b_{i}))]} \nonumber \\= & {} 1-\Psi _{i}, \end{aligned}$$

with $\Psi$ the logistic function in (2). Alternatively, the change in the $\gamma _{i}$ parameters can be traded off by

28 $$\begin{aligned} a_{i}^{*}=\ln (\kappa [1+\exp (a_{i}(\theta -b_{i}))]-1]/(\theta -b_{i})>0, \end{aligned}$$

or

29 $$\begin{aligned} \theta ^{*}=b_{i}+\ln (\kappa [1+\exp (a_{i}(\theta -b_{i}))]-1]/a_{i} \end{aligned}$$

where the nonnegativity requirement for $a_{i}^{*}$ implies both $$\kappa >2(1-\Psi )\ $$ and $\theta >b_{i}$. $\square$

The status of the $c_{i}$ parameters in the version of the 3PL model with all parameters free is still unknown. But the last two cases are already enough to illustrate the problematic role of these parameters, which has largely been ignored in the literature. Lord (1980, pp. 36, 184–185) even claims that the $c_{i}$ parameters are actually identifiable. As already noted, an exception was Maris (2002), who introduced the second case above.

**Fig. 1:**
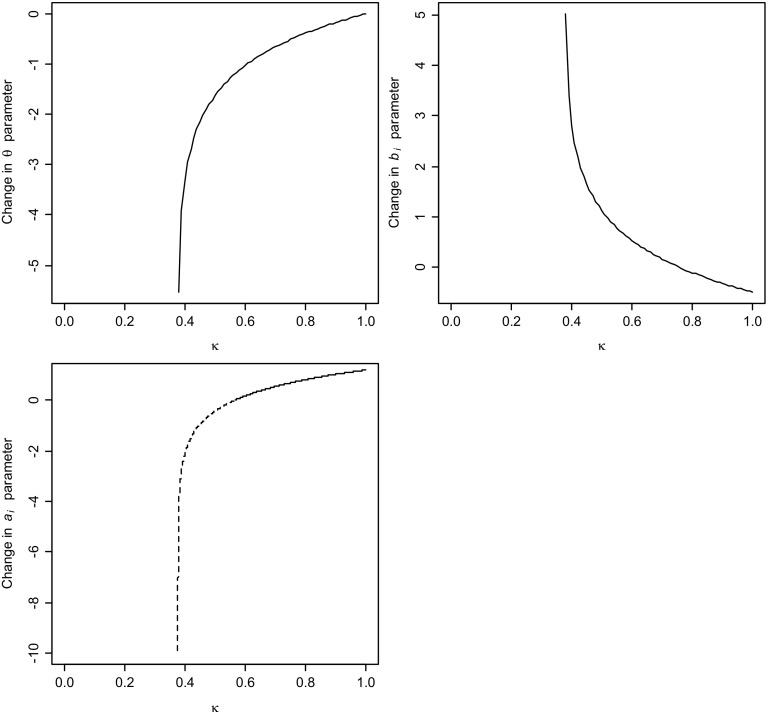
Change in $\theta$, $b_i$, and $a_i$ parameters compensating the change in $\gamma _i=1-c_{\mathrm{i}}$ by a factor *k*, for an item with $a_i=1$ and $b_i=-0.5$ and the ability parameter fixed at $\theta =0$. *Dashed line* represents negative values for the $a_i$ parameter.

For the third case, Fig. 1 illustrates how dramatic the tradeoff between the $$\gamma _{i}$$ and $$a_{i}$$, $$b_{i}$$  and $$\theta $$ parameters can be. It displays each of the tradeoffs as a function of $$\kappa $$ for an item with $$ a_{i}=1.0$$, $$b_{i}=-.5$$  and common ability parameter $$\theta =0$$. For these parameter values, $$1-\Psi =.378$$; hence, the range of admissible values for $$ \kappa $$ is (.378, 1] for the functions in (26) and (29) but (.755, 1] for the one in (28). Observe that $$\kappa =1$$ means no change in the $$\gamma _{i}$$ parameter; for smaller values of $$\kappa $$, $$ \gamma _{i}$$ decreases in size (and $$c_{i}$$ thus becomes larger). The change in the $$b_{i}$$ and $$\theta $$ parameters as a function of the decrease in $$\gamma _{i}$$ is remarkable, especially closer to their vertical asymptote at $$\kappa =.378.$$ On the other hand, the $$a_{i}$$ parameter appears to be quite robust across its range of admissible values for $$\kappa $$. The fact that the tradeoff between the lower asymptote and the slope of the response function seems much less dramatic than for the other two parameters might go against our intuition, but is entirely due to the nonnegativity requirement for the slope parameter. If we did admit negative values for it, the slope parameter could go down, for example, all the way to $$a_{i}^{*}=-10$$ for $$\kappa =.38$$ (just above the vertical asymptote).

The tradeoffs in (3)–(5) imply lack of identifiability of all parameters in the 2PL model and 1PL/Rasch model. Wood (1978) analyzed a special version of the Rasch model for equivalent items with success probability

30 $$\begin{aligned} \pi _{pi}\equiv \frac{\exp (\theta _{p})}{1+\exp (\theta _{p})}, \end{aligned}$$

for all *i*. This “0PL model” is just a reparameterization of the Bernoulli probability function in (19 ). It is thus fully identifiable (although unlikely to show any satisfactory fit to the items a real-world testing program).

The literature offers only a few examples of sets of identifiability restrictions for special cases of the 3PL model in (1)–(2 ) for which sufficiency has formally been proven. For instance, for the 1PL/Rasch model, as is well known, it is sufficient to set the difficulty parameter of one item or the ability parameter of one test taker equal to a known constant. Equivalently, we could impose a linear constraint on a subset of these parameters (e.g., constrain their mean to a known value). As shown by San Martín et al. (2009) for the 1PL-G model (i.e., 1PL/Rasch model extended with a guessing parameter for each item), it is sufficient to fix the difficulty and guessing parameters of one item to known constants. Recently, the same authors have shown that, contrary to what one might have expected intuitively, it is not sufficient to fix all three parameters of an arbitrary item to known constants to make the (fixed-effects) 3PL model in (21) identifiable (San Martín, Gonzáles, & Tuerlinckx, 2014). In fact, we are not aware of any existing proof of a set of identifiability restrictions sufficient for it. In the absence of such proofs (but the presence of large amounts of response data), the practical solution in the testing industry has been to circumvent the problem using a two-stage calibration procedure. In its first stage, the ability parameters are temporarily treated as a random sample from a population distribution, which allows for marginalization of the likelihood for the fixed-effects version of the model with respect to an assumed ability distribution and maximum marginal likelihood (MML) estimation of all item parameters. The second stage consists of subsequent maximum likelihood or Bayesian (e.g., EAP) estimation of the individual ability parameters assuming the item parameters have been estimated from enough response data to treat them as known. Typically, the ability distribution in the first stage is taken to be the standard normal, which implies the adoption of (12) as *de facto* standard set of identifiability restrictions for the 3PL model in the field of educational testing. Although its effectiveness has been confirmed in the daily practice of item calibration (e.g., convergence of parameter estimates, which would have been problematic with lack of identifiability) as well as through numerous parameter recovery studies, we still await a formal proof of its sufficiency. As for the second stage, if the item parameters can be treated as known, (21) defines known monotonic relationships between each of the ability parameters $\theta _{p}$ and their success parameters $\pi _{pi}$ , and the former are therefore identified as well.

For all practical purposes, the use of (12) in this two-stage detour thus restricts the parameters in the specification of the 3PL model in (21) to fixed values (to which the MML estimators of the item parameters and the subsequent estimates of the ability parameters converge with the sample size and test length, respectively). The reverse does not necessarily hold, though; identifiability of a fixed-effect specification does not automatically imply the same for its random-effect specification (San Martín, Rolin, et al. 2013).

## Linking Functions

The main goal of this section is to define the problem of linking IRT parameters from different calibration studies and derive the specific linking functions necessary for the 3PL model. More specifically, we address the problem of post hoc linking; that is, mapping the parameters from one study onto the values they would have had if they had been included in another *after* both calibrations have been conducted. This type of linking is common in the testing industry. As both the item and ability parameters are to be linked simultaneously, the linking automatically is for the fixed-effects specification of the 3PL model. In principle, it is possible to avoid the problem by concurrent recalibration of the response data collected in different studies, capitalizing on the presence of common items or test takers in them and imposing constraints on the parameters. For this approach, it has even been proposed to tentatively impose constraints, e.g., linear constraints as in (3)–(5) and check on their appropriateness using Lagrange multiplier tests (von Davier & von Davier, 2011). But such strategies are not always practical for testing programs that have to link their parameters continuously across multiple test administrations.

Our current treatment of the linking problem only deals with its mathematical aspects at the level of the model parameters, without any bothering about the fact that these parameters are unknown. In order to actually use them in practice, linking functions need to be estimated from response data. But we are only able to find defendable estimators and evaluate their statistical quality once we have an explicit definition of their estimand.

We begin with considering the more general case of a parametric response model used to calibrate the responses for a set of *P* test takers on *I* items with the vector of success probabilities ${\varvec{\pi }}=(\pi _{pi})$ in (20). Let $f(\cdot )$ be the response function specified by the model, ${\varvec{\xi }}_{pi}$ its vector of parameters for the combination of test taker *p* and item *i*,  and ${\varvec{\xi }} =({\varvec{\xi }}_{pi})$ the vector of parameters for all test takers and items. The choice of model amounts to the adoption of a system of $P\times I$ equations $\pi _{pi}=f({\varvec{\xi }}_{pi})$. As the probabilities $\pi _{pi}$ are identified and thus have fixed values for all combinations of *p* and *i*, each of the equations introduces a level surface (contour) in the domain of *f*, which is the subset of all values of ${\varvec{\xi }}$ for which $f({\varvec{\xi }}_{pi})=\pi _{pi}$ is true. The solution set for the system of equations is the intersection of all $P\times I$ surfaces. As the system lacks identifiability of the model parameters, the set consists of more than one point. Identifiability restrictions are extra equations added to the system. The intersection of their solution sets with the set for the system reduces the latter to a unique point, whose coordinates are the true values of the item and test taker parameters for the calibration.

Now, suppose we have conducted two separate calibration studies that had both unique and common test takers and/or items in them. Both studies are assumed to have used appropriate sets of identifiability restrictions. Obviously, the use of different restrictions implies different intersections of their solution sets with those determined by the two systems of model equations, and hence different true values for the common parameters in the two calibrations. But different true values can also arise if formally identical sets of identifiability restrictions have been imposed on the two calibrations. The presence of unique test takers and/or items in the calibrations implies different vectors of success probabilities ${\varvec{\pi }}^{*}\ne$ ${\varvec{\pi }}$ for them and thus different solutions sets for their model equations. Consequently, their intersections with the solution set for the identifiability restrictions generally differ, and the same items or test takers assigned to the two calibrations can therefore have different true parameter values. The critical factor is the scope of the restrictions. For example, if they fix the values of some of the common parameters to the same known constants in the two calibrations, obviously their impact on them is identical. But if they include unique parameters and leave the common parameters free, they yield different true values for the latter—an observation confirmed by the educational testing industry, where invariably different parameter values are found for common parameters in separate calibrations with large samples of test takers for the restrictions in (13) and (14), as well as by our example later in this paper. In fact, if this differential effect did not exist, we would not have to link any parameters.

Consider a hypothetical combination of a test taker and item assigned to two of these calibration studies with identified parameters. Let ${\varvec{\xi }} ^{*}$ and ${\varvec{\xi }}$ denote the vectors with the unique true values for the combination in the calibrations (where the indices have been omitted for notational convenience, as well as to emphasize the hypothetical nature of the combination). For example, for the 3PL model, ${\varvec{\xi }} ^{*}=(\theta ^{*},a^{*},b^{*},c^{*})$ and ${\varvec{\xi }} =(\theta ,a,b,c)$. The question of how to map ${\varvec{\xi }}$ and ${\varvec{\xi }}^{*}$ onto one another is the topic of this section. Observe that, although different, both ${\varvec{\xi }}^{*}$ and ${\varvec{\xi }}$ are associated with the *same* success probability $\pi$ for the combination of test taker and item. This fact is key in our derivation of the mapping below.

Our first theorem is for a general response model that specifies success probability $\pi$ for each combination of a test taker and item only as a monotone continuous function of their parameters, where the monotonicity is taken to mean that $\pi$ is strictly increasing or decreasing in each of the components of ${\varvec{\xi }}$ with all other components fixed at any of their admissible values. We then present our results for the 3PL model. The version of this model for the regular parameterization in (1)–(2) is both continuous and monotone in each of its parameters, provided we exclude the case of $\theta =b$ for the *a* parameter; the version with the slope-intercept parameterization is monotone in each of its parameters without any further restriction.

Again, except for an illustrative example below, the current paper only deals with the mathematical aspects of linking functions; the problem of how to actually estimate them and evaluate their estimation error deserves separate treatment.

### **Theorem 3**

Assume a response model with a fixed parameter structure that (i) specifies $\pi$ as a monotone continuous function of its parameters and (ii) has been used in two separate calibration studies with identified parameters ${\varvec{\xi }}$ and ${\varvec{ \xi }}^{{*}}$. Then ${\varvec{\xi }}^{*}$ is linked to ${\varvec{ \xi }}$ by a vector function

31 $$\begin{aligned} {\varvec{\xi }}^{*}\mathbf {=}\varphi ({\varvec{\xi }})\mathbf {=(}\varphi _{1}(\xi _{1}),\ldots ,\varphi _{d}(\xi _{d})) \end{aligned}$$

with components $\varphi _{1},\ldots ,\varphi _{d}$ that are both monotone and continuous.

### *Proof*

Let $\xi ^{*}$ and $\xi$ be an arbitrary pair of corresponding components of ${\varvec{\xi }}^{*}$ and ${\varvec{\xi }}$ **.** Fixing all other components, the monotonicity of the model implies the existence of monotone functions $\pi \mathbf {=}f(\xi ^{*})$ and $\pi \mathbf {=}g(\xi ).$ Hence, there also exists a function $\xi ^{*}=f^{-1}(g(\xi ))=\varphi (\xi )$. Being a composite of continuous functions, $\varphi$ is continuous. Further, as both *f* and *g* are bijective, $\varphi$ is bijective as well. Suppose that $\varphi$ is not monotone. It then has an interior point $\xi _{0}$ in its domain with a local optimum. But this implies the existence of points $\xi ^{\prime }<\xi _{0}<$ $\xi ^{\prime \prime }$ with $\varphi (\xi ^{\prime })=\varphi (\xi ^{\prime \prime })$, which contradicts the fact that $\varphi$ is bijective. Thus, $\varphi$ is monotone. $\square$

The feature of monotonicity should not come as a surprise. If it did not hold, the two sets of the identifiability restrictions would imply a different order of some of the parameters, for instance, a reversal of the difficulties of two items, which is impossible without violating the requirement of observational equivalence. Observe, however, that $\varphi ({\varvec{\xi }})$ is only required to be *componentwise *monotone; it does not need to hold that all components be increasing or all of them be decreasing.

It is thus possible to view the impact of the use of different sets of identifiability restrictions as a componentwise reparameterization of the response model, which leaves the structure of the model intact. However, unlike the earlier case of a known function in (16) applied to unknown parameter values, we now have to address the reverse problem: This time the two sets of parameter values are given, and we have to find the componentwise bijective function that maps them onto one another. Observe again that the specific identifiability restrictions used in the calibrations need not be known at all; neither do we need to assume anything about the statistical estimation method through which the restrictions might have been imposed. Only their impact on the item and test taker parameters counts.

For the 3PL model, the linking function ${\varvec{\varphi }}\mathbf {=(}\varphi _{\theta }(\theta ),\varphi _{a}(a),\varphi _{b}(b),\varphi _{c}(c))$ has to be derived from (1)–(2) for two arbitrary sets of values ($\theta ^{*},a^{*},b^{*},c^{*}$) and ($\theta ,a,b,c$). However, it is simpler to use the first equation in (24), and find $\varphi _{\theta }$, $\varphi _{a}$, $\varphi _{b}$, and $\varphi _{\gamma }$ as the solution of

32 $$\begin{aligned} \frac{\varphi _{\gamma }(\gamma )}{1+\exp [\varphi _{a}(a)(\varphi _{\theta }(\theta )-\varphi _{b}(b))]}=\frac{\gamma }{1+\exp [a(\theta -b)]}, \end{aligned}$$

with additional back transformation of $\varphi _{\gamma }$ to $\varphi _{c}$ . The required linking function is thus the solution of a functional equation in four unknowns (for relevant theory of functional equations, see, for instance, Sahoo & Kannappan, 2011, or Small, 2007). The next theorem shows the solution:

### **Theorem 4**

Given the conditions in Theorem  3), the linking function for the 3PL model is

33 $$\begin{aligned} \varphi _{a}(a)= & {} u^{-1}a, \end{aligned}$$

34 $$\begin{aligned} \varphi _{b}(b)= & {} ub+v, \end{aligned}$$

35 $$\begin{aligned} \varphi _{c}(c)= & {} c, \end{aligned}$$

and

36 $$\begin{aligned} \varphi _{\theta }(\theta )=u\theta +v, \end{aligned}$$

with

37 $$\begin{aligned} u\equiv \frac{\varphi _{\theta }(\theta )-\varphi _{b}(b)}{\theta -b},\text { }\theta \ne b, \end{aligned}$$

and

38 $$\begin{aligned} v=\varphi _{b}(b)-ub=\varphi _{\theta }(\theta )-u\theta . \end{aligned}$$

### *Proof*

From (32),

39 $$\begin{aligned} \varphi _{\gamma }(\gamma )=\frac{1+\exp [\varphi _{a}(a)(\varphi _{\theta }(\theta )-\varphi _{b}(b))]}{1+\exp [a(\theta -b)]}\gamma . \end{aligned}$$

As this function is monotone in $\gamma$, $\varphi _{\gamma }$ is a monotone component of ${\varvec{\varphi }}$, and therefore

40 $$\begin{aligned} \frac{1+\exp [\varphi _{a}(a)(\varphi _{\theta }(\theta )-\varphi _{b}(b))]}{ 1+\exp [a(\theta -b)]}=\kappa >0, \end{aligned}$$

is a constant independent of $\gamma$. Thus, $\varphi _{\gamma }(\gamma )=\kappa \gamma .$ However, since $\varphi _{\gamma }$ is a monotone mapping from [0, 1] onto itself, $\kappa =1$ and (35) follows. We now have to find $\varphi _{\theta }$, $\varphi _{a}$, and $\varphi _{b}$ as the solution of (40) for $\kappa =1$; that is,

41 $$\begin{aligned} \varphi _{a}(a)[\varphi _{\theta }(\theta )-\varphi _{b}(b)]=a(\theta -b) \text {.} \end{aligned}$$

Rewriting the equation,

42 $$\begin{aligned} \varphi _{a}(a)=\frac{\theta -b}{\varphi _{\theta }(\theta )-\varphi _{b}(b)} a, \end{aligned}$$

with $\varphi _{\theta }(\theta )\ne \varphi _{b}(b)$. But, as $\varphi _{a}$ is a @@@monotone@@@ component of ${\varvec{\varphi }}$ as well ,

43 $$\begin{aligned} \frac{\varphi _{\theta }(\theta )-\varphi _{b}(b)}{\theta -b}=\text {const,} \end{aligned}$$

which is our key equation. First, (33) follows directly from (43) along with the definition of its constant in (37). Further, (43) shows that $\varphi _{\theta }(x)-\varphi _{b}(x)$ is equal to a constant times $\theta -b$. Substituting $x=\theta =b$ yields $\varphi _{\theta }(x)-\varphi _{b}(x)=0,$ and it thus holds that $\varphi _{\theta }=\varphi _{b}=\varphi$. Observe also that (43) implies a constant difference quotient for $\varphi$. Hence, $\varphi$ is linear, and (34) and (36) hold. Finally, (38) follows from (34)–(36). $\square$

Although the proof did not make any assumptions as to the general shape of the functions that map the values of the $a_{i}, b_{i},$ and $\theta _{p}$ parameters in a new calibration onto those in an earlier calibration, they appear to be linear, just as currently assumed in the literature; see our review in (3)–(5). However, a new result is the definition of linking parameter *u*, and consequently of *v*. Unlike (6)–(8), (37) defines it as the ratio of the differences between the test taker’s ability and the difficulty of the item in the two calibrations. The reason for the difference between these new and old definitions may be the failure in the current literature to distinguish between the formal definitions of *u* and *v* and their solutions from the system of linking equations implied by the choice of linking design. As demonstrated in the next section, separating the two does give us large flexibility to derive alternative solutions for *u* and *v* from alternative designs. Another new result is the derivation of the identity function for the $c_{i}$ parameters @@@. We will further reflect on its practical implications in the last section of this paper.

In addition, it is important to note the different nature of these functions for the four different types of parameters. The one for the $$c_{i}$$ parameters is an identity function, which does not involve either of the linking parameters *u* and *v*. On the other hand, the function for the $$ a_{i}$$ parameters involves one linking parameter, *u*, whereas those for the $$b_{i}$$ and $$\theta _{p}$$ parameters depend both on *u* and *v*. Thus, unlike the $$c_{i}$$ parameters, the latter can be linked only when the numerical values of the linking parameters are known. This obvious point takes us to another identifiability requirement, namely for the system of linking equations to be derived from (33)–(38) for the specific design adopted in the linking study.

## Identification of Linking Parameters

Basically, a linking design is a combination of two calibration designs with common items and/or test takers. Once it has been selected, (33)–(38) can be used to derive a new system of equations of the unknown linking parameters *u* and *v* in the parameter values for the common items or test takers in the two calibrations. Of course, *u* and *v* have unique values only when the system is identified. The problem of linking item response model parameters thus involves three different types of identifiability requirements, two for the system of the model equations in (21) associated with the two calibrations and another for the system of linking equations that is used. At this stage, the former have already been met through the adoption of extra restrictions in the two calibrations. The latter, although sometimes critical (e.g., Theorem 7 below), involves only an appropriate choice of linking design; no additional restrictions are necessary.

We illustrate the process for three minimal designs. In doing so, $p=1,\ldots ,n$ and $i=1,\ldots ,m$ and are now used as indices for the common persons and items in the design, respectively, while $t=1,2$ will be used to denote the two calibrations. Thus, $(a_{i_{t}},b_{i_{t}})$ and $\theta _{p_{t}}$ are the true values of the pertinent parameters for item *i* and the parameter for test taker *p* in the *t*th calibration, respectively. Because the linking function for the $c_{i}$ parameters is already know, these parameters can further be ignored.

### One Common Item

Linking parameters *u* and *v* are already identified if the two calibrations have one common item, $i=1$. The system of linking equations then follows from (33) and (38) as

44 $$\begin{aligned} u= & {} \frac{a_{1_{1}}}{a_{1_{2}}},\text { }a_{1_{2}}>0, \end{aligned}$$

45 $$\begin{aligned} v= & {} b_{1_{2}}-ub_{1_{1}}. \end{aligned}$$

### Two Common Items

For a pair of common items $i=1,2,$ we could use (44)–(45) for either of them. But now an alternative is available in the form of the substitution of their two sets of parameter values ($b_{1_{1}},b_{2_{1}}$) and ($b_{1_{2}},b_{2_{2}}$), $b_{1_{1}}\ne b_{2_{1}}$, into (38). Elimination of *v* then gives

46 $$\begin{aligned} u=\frac{b_{1_{2}}-b_{2_{2}}}{b_{1_{1}}-b_{2_{1}}}, \end{aligned}$$

whereupon *v* is equal to

47 $$\begin{aligned} v=b_{i_{2}}-ub_{i_{1}}, \quad i=1,2. \end{aligned}$$

This simple system of equations gives unique values for the linking parameters once their item parameters are identified. A similar property does not hold for the next type of design.

### One Common Test Taker

It appears to be impossible to derive a system of equations from (33) and (38) for a common test taker with ($\theta _{1_{1}},\theta _{1_{2}}$) from which *u* and *v* are identifiable.

### Two Common Test Takers

For a pair of test takers with $\theta _{1_{1}}\ne \theta _{2_{1}}$, *u* and *v* can be obtained as

48 $$\begin{aligned} u=\frac{\theta _{1_{2}}-\theta _{2_{2}}}{\theta _{1_{1}}-\theta _{2_{1}}}, \end{aligned}$$

and

49 $$\begin{aligned} v=\theta _{p_{2}}-u\theta _{p_{1}},\quad p=1,2. \end{aligned}$$

Because of their practical importance, we document these results as a theorem:

#### **Theorem 5**

For the 3PL model with standard parameterization, linking parameters *u* and *v* are already identifiable for a common-item design with at least one common item and a common-person design with at least two common test takers.

As a typical linking study has more than these minimal numbers of items or test takers, we easily have multiple systems of linking equations, each returning the same unique values for the linking parameters *u* and *v* . At the current level of true parameter values, any choice from them would thus suffice. However, in practice, except when some of common item or ability parameters were fixed at known constants, in which case we can just substitute these constants into (44)–(49), all parameters are estimated. A first suggestion of how to combine estimates of *u* and *v* from multiple systems of linking equations as in (44)–(49) is offered in the empirical example below.

### Slope-Intercept Parameterization

Earlier we wondered what the impact of a change of parameter structure on the linking function would be. Theorem 6 highlights the impact for the slope-intercept parameterization for the 3PL model.

#### **Theorem 6**

Given the conditions in Theorem 3, the linking function for the slope-intercept parameterization $\alpha \vartheta +\beta$ of the 3PL model is

50 $$\begin{aligned} \varphi _{\alpha }(\alpha )= & {} (\alpha -u)/v,\quad v>0, \end{aligned}$$

51 $$\begin{aligned} \varphi _{\beta }(\beta )= & {} \beta +u, \end{aligned}$$

52 $$\begin{aligned} \varphi _{c}(c)= & {} c \end{aligned}$$

and

53 $$\begin{aligned} \varphi _{\vartheta }(\vartheta )=(\vartheta -u)/w,\quad w\ne 0, \end{aligned}$$

with

54 $$\begin{aligned} u=\varphi _{\beta }(0),v=\varphi _{\vartheta }(1)\text {, and }w=\varphi _{\alpha }(1). \end{aligned}$$

#### *Proof*

The component for the linking of *c* does not change because these parameters are left untouched by the reparameterization. But we now have to find $\varphi _{\xi }$, $\varphi _{\alpha }$, and $\varphi _{\beta }$ as the solution of

55 $$\begin{aligned} \varphi _{\alpha }(\alpha )\varphi _{\vartheta }(\vartheta )+\varphi _{\beta }(\beta )=\alpha \vartheta +\beta , \end{aligned}$$

or, equivalently,

56 $$\begin{aligned} \varphi _{\beta }(\beta )=\beta +\alpha \vartheta -\varphi _{\alpha }(\alpha )\varphi _{\vartheta }(\vartheta ). \end{aligned}$$

Substituting $$\beta =0$$ yields

57 $$\begin{aligned} \alpha \vartheta -\varphi _{\alpha }(\alpha )\varphi _{\vartheta }(\vartheta )=u, \end{aligned}$$

with *u* given by (54). Hence, (51) follows from (56 )–(57). Likewise, substituting $\vartheta =1$ into (57), we obtain (50) with *v* given by (54), while substitution of $\alpha =1$ leads to (53). $\square$

Although still linear, the linking functions in (50)–(54) differ considerably from those for the regular parameterization of the 3PL model; in fact, they now appear to have three rather than two unknown parameters. More importantly, an attempt to derive an identified system of linking equations from them appears to run into practical problems. Still assuming all model parameter to be known, (54) suggests selecting a common item with $\beta _{i_{1}}=0$ and $\alpha _{i_{1}}=1$ and common test taker with $\vartheta _{p_{1}}=1$ for the first calibration and equating the three linking parameters to their values in the second calibration, that is, setting $u=\beta _{i_{2}},$ $w=\alpha _{i_{2}},$ and $v=\vartheta _{p_{2}}$. However, the presence of items and test takers with such exact parameter values in a linking study is highly unlikely.

An alternative seems to solve (50)–(53) for *u*,  *v*, and *w* , obtaining them as

58 $$\begin{aligned} u= & {} \beta _{i_{2}}-\beta _{i_{1}} \end{aligned}$$

59 $$\begin{aligned} v= & {} \frac{\alpha _{i_{1}}-u}{\alpha _{i_{2}}},\quad \alpha _{i_{2}}\ne 0, \end{aligned}$$

and

60 $$\begin{aligned} w=\frac{\vartheta _{p_{1}}-u}{\vartheta _{p_{2}}},\quad \vartheta _{p_{2}}\ne 0. \end{aligned}$$

for an arbitrary *p* and *i*. However, we now need a linking design with both at least one common item for (58)–(59 ) and one common test taker for (60), a new requirement entirely due to the change in parameter structure created by (15). Hence the following theorem:

#### **Theorem 7**

For the 3PL model with slope-intercept parameterization, linking parameters *u*, *v*, and *w* are only identifiable for designs with both common test takers and common items.

The result in this theorem has a major practical implication. If one believes that IRT models always have a scale for the person parameter with an arbitrary unit and zero, as the literature referenced in our introductory section appears to do, it may seem natural to adopt the same linking functions as for the model with the standard parameterization in (33)–(38). This choice is incorrect; the proper functions are those in (58)–(60). However, we do not expect the latter to be practised regularly as they require a linking design with test takers responding twice but independently to the same items—an assumption unlikely ever to be met because of memory effects. An alternative would be to transform the estimated slope and intercept parameters back to the standard parameters and link the latter, but then we miss the covariance matrices for the estimators of the model parameters necessary to evaluate the standard errors of linking as in the example in the next section.

## Illustrative Example

Although the focus of this paper was not yet on a statistical treatment of the linking problem, a small example might already illustrate some of the practical consequences of our theoretical results. The example is for a common-item design for the 3PL model in its standard parameterization. The design allows us to use (44)–(45) to estimate linking parameters *u* and *v* in (33)–(38) for the two calibrations.

Response data were generated for two test forms each existing of 20 unique and 20 common items. All common items had $c_{i}=.25$, while their $a_{i}$ and $b_{i}$ parameters were chosen to represent one of the possible combinations of $b_{i}=-2(.5)2$ with $a_{i}=.5,$ 1.5,  using $b_{i}=0$ twice to get a total of 20 items. All unique items had $c_{i}=.25$ as well, but their $a_{i}$ and $b_{i}$ parameters were randomly sampled from *U*(.5, 2 ) and *N*(0, 1), respectively. The first calibration had ability parameters for 10,000 test takers sampled from $N(-.5,2)$; for the second calibration, the parameters for 10,000 test takers were sampled from *N*(.5, 1.5). The item parameters for the two test forms were estimated separately using the *MIRT Scaling Program*, version 1.0 (Glas, 2010), with MML estimation with $\theta \sim N(0,1)$ in both runs.

Table 1 contains the generating and estimated values of the common item parameters. Observe that even though the response data were generated for exactly the same sets of parameters for the common items, the parameters of the common items in the two calibrations were estimated to be quite different. As argued in our earlier discussion of (13)–(14), the reason is the different effect of the $\theta \sim N(0,1)$ restriction on all parameter values in the presence of test takers with different abilities in the two calibrations.

**Table 1:** Generating and estimated parameter values for the common items.

Common	Generating values			Calibration 1			Calibration 2		
item	$$ a_{i}$$	$$ b_{i}$$	$$ c_{i}$$	$$ \widehat{a}_{i}$$	$$\widehat{b}_{i}$$	$$\widehat{c}_{i}$$	$$\widehat{a}_{i}$$	$$\widehat{b}_{i}$$	$$\widehat{c}_{i}$$
1	1.500	$-2.000$	0.250	2.612	$-0.843$	0.213	2.162	$-1.725$	0.191
2	1.500	$-1.500$	0.250	2.707	$-0.558$	0.234	2.334	$-1.333$	0.212
3	1.500	$-1.000$	0.250	2.612	$-0.297$	0.232	2.358	$-0.959$	0.29
4	1.500	$-0.500$	0.250	2.751	$-0.008$	0.241	2.125	$-0.728$	0.228
5	1.500	0.000	0.250	2.83	0.283	0.258	2.14	$-0.364$	0.238
6	1.500	0.000	0.250	2.722	0.296	0.264	2.283	$-0.338$	0.248
7	1.500	0.500	0.250	2.596	0.536	0.241	2.013	$-0.046$	0.233
8	1.500	1.000	0.250	2.506	0.773	0.231	2.183	0.33	0.253
9	1.500	1.500	0.250	3.15	1.09	0.249	2.478	0.663	0.257
10	1.500	2.000	0.250	2.605	1.331	0.246	2.176	1.004	0.249
11	0.500	$-2.000$	0.250	1.022	$-0.652$	0.295	0.745	$-1.518$	0.284
12	0.500	$-1.500$	0.250	0.897	$-0.523$	0.245	0.685	$-1.486$	0.256
13	0.500	$-1.000$	0.250	0.86	$-0.51$	0.171	0.698	$-1.071$	0.234
14	0.500	$-0.500$	0.250	0.854	$-0.209$	0.181	0.715	$-0.857$	0.187
15	0.500	0.000	0.250	0.938	0.311	0.246	0.77	$-0.447$	0.225
16	0.500	0.000	0.250	0.904	0.205	0.233	0.715	$-0.455$	0.228
17	0.500	0.500	0.250	0.971	0.497	0.244	0.647	$-0.276$	0.173
18	0.500	1.000	0.250	0.924	0.841	0.248	0.762	0.248	0.227
19	0.500	1.500	0.250	0.946	1.087	0.245	0.655	0.562	0.229
20	0.500	2.000	0.250	0.826	1.352	0.225	0.647	0.86	0.216

Table 2 summarizes the covariance matrices for the MML estimators for each of the common items produced by the scaling program. The data in this table are needed to evaluate the estimates of the parameters for the linking function between the two calibrations. Observe that the variances for the item parameter estimates are as expected for datasets of the current size and generating parameter values.

**Table 2:** Estimated (co)variances for the estimators of the common item parameters.

Common item	Calibration 1						Calibration 2					
	$\widehat{\sigma }_{a_{i}}^{2}$	$\widehat{\sigma } _{b_{i}}^{2}$	$\widehat{\sigma }_{c_{i}}^{2}$	$\widehat{\sigma } _{a_{i}b_{i}}$	$\widehat{\sigma }_{a_{i}c_{i}}$	$\widehat{\sigma } _{b_{i}c_{i}}$	$\widehat{\sigma }_{a_{i}}^{2}$	$\widehat{\sigma } _{b_{i}}^{2}$	$\widehat{\sigma }_{c_{i}}^{2}$	$\widehat{\sigma } _{a_{i}b_{i}}$	$\widehat{\sigma }_{a_{i}c_{i}}$	$\widehat{\sigma }_{b_{i}c_{i}}$
1	0.023	0.001	0.002	0.001	0.006	0.001	0.019	0.004	0.012	0.002	0.012	0.005
2	0.020	0.001	0.001	0.001	0.003	0.001	0.017	0.002	0.003	0.000	0.006	0.001
3	0.015	0.001	0.000	0.001	0.002	0.000	0.015	0.001	0.001	0.000	0.003	0.001
4	0.015	0.001	0.000	0.002	0.001	0.000	0.009	0.001	0.001	0.001	0.002	0.001
5	0.018	0.001	0.000	0.004	0.001	0.000	0.010	0.001	0.000	0.002	0.002	0.001
6	0.017	0.001	0.000	0.004	0.001	0.000	0.011	0.001	0.000	0.001	0.001	0.000
7	0.015	0.002	0.000	0.004	0.001	0.000	0.009	0.001	0.000	0.002	0.001	0.001
8	0.016	0.003	0.000	0.006	0.001	0.000	0.014	0.002	0.000	0.004	0.001	0.000
9	0.044	0.006	0.000	0.016	0.001	0.000	0.020	0.003	0.000	0.007	0.001	0.000
10	0.033	0.009	0.000	0.016	0.001	0.000	0.022	0.006	0.000	0.011	0.001	0.001
11	0.008	0.036	0.006	0.015	0.006	0.014	0.009	0.215	0.031	0.039	0.015	0.081
12	0.007	0.053	0.007	0.018	0.006	0.019	0.009	0.292	0.036	0.047	0.017	0.103
13	0.007	0.055	0.008	0.017	0.007	0.021	0.008	0.180	0.021	0.034	0.012	0.061
14	0.006	0.048	0.005	0.016	0.005	0.016	0.007	0.125	0.015	0.027	0.009	0.043
15	0.008	0.032	0.002	0.014	0.003	0.008	0.007	0.073	0.007	0.020	0.006	0.022
16	0.007	0.036	0.002	0.014	0.004	0.009	0.007	0.109	0.009	0.026	0.008	0.031
17	0.007	0.027	0.001	0.013	0.003	0.006	0.007	0.152	0.011	0.031	0.008	0.040
18	0.008	0.036	0.001	0.016	0.003	0.006	0.008	0.066	0.004	0.021	0.005	0.015
19	0.009	0.035	0.001	0.017	0.002	0.005	0.009	0.136	0.005	0.033	0.006	0.025
20	0.010	0.062	0.001	0.023	0.003	0.008	0.010	0.139	0.004	0.034	0.006	0.023

We know that the $c_{i}$ parameters in the two calibrations are linked by the identity transformation. Thus, for example, if we needed to know the value of the $c_{i}$ parameter that an arbitrary items in the first calibration would have had in the second calibration, we could just use $\widehat{c}_{i}$ obtained in the first calibration as its estimate. (For the common items, it makes more sense to pool their two estimates, though.) For the other parameters, we need to know the linking parameters *u* and *v*, which can be estimated simply by plugging $\widehat{a}_{i}$ and $\widehat{b}_{i}$ for each common item into (44)–(45). The results are shown in Table 3. Although each of these 20 estimates reveals the same trend, they show considerable random variation. In order to evaluate the variation, Table 3 also gives the estimated standard errors for each $\widehat{u}_{i}$ and $\widehat{v}_{i}$, which were derived from the (co)variances for $a_{i}$ and $b_{i}$ in Table 2 using the (first-order) multivariate delta method (e.g., Casella & Berger, 2002, sect. 5.5.4). As the size of these standard errors indicate, we should have expected a considerable amount of variation indeed.

**Table 3:** Linking parameter and their standard errors estimated for each common item.

Common item	$\widehat{u}_{i}$	$\widehat{\sigma }_{u_{i}}$	$\widehat{v}_{i}$	$\widehat{\sigma }_{v_{i}}$
1	1.208	0.105	$-0.707$	0.104
2	1.160	0.088	$-0.686$	0.069
3	1.108	0.077	$-0.630$	0.045
4	1.295	0.082	$-0.718$	0.047
5	1.322	0.088	$-0.738$	0.074
6	1.192	0.079	$-0.691$	0.069
7	1.290	0.086	$-0.737$	0.106
8	1.148	0.084	$-0.557$	0.134
9	1.271	0.111	$-0.723$	0.228
10	1.197	0.117	$-0.589$	0.287
11	1.372	0.210	$-0.624$	0.413
12	1.309	0.218	$-0.801$	0.516
13	1.232	0.194	$-0.443$	0.425
14	1.194	0.178	$-0.607$	0.406
15	1.218	0.171	$-0.826$	0.396
16	1.264	0.191	$-0.714$	0.444
17	1.501	0.238	$-1.022$	0.573
18	1.213	0.184	$-0.772$	0.490
19	1.444	0.256	$-1.008$	0.724
20	1.277	0.247	$-0.866$	0.811

Obviously, as each $\widehat{u}_{i}$ and $\widehat{v}_{i}$ is an estimate of the same *u* and *v*, respectively, rather than using them individually, they should be combined into overall estimates. A natural suggestion is to use their precision-weighted average, with the inverse of their squared standard errors, $\sigma _{u_{i}}^{-2}$ and $\sigma _{v_{i}}^{-2},$ as measure of precision. The estimator of *u* is then

61 $$\begin{aligned} \widehat{u}=\left( \sum \limits _{i=1}^{20}\widehat{\sigma }_{u_{i}}^{-2} \widehat{u}_{i}\right) \Bigg / \left( \sum \limits _{i=1}^{20}\widehat{\sigma } _{u_{i}}^{-2}\right) , \end{aligned}$$

with estimated standard error

62 $$\begin{aligned} \widehat{\sigma }_{u}=\left( \sum \limits _{i=1}^{20}\widehat{\sigma } _{u_{i}}^{-2}\right) ^{-1/2}, \end{aligned}$$

with similar expressions for the estimator of *v*.

Table 4 shows these overall estimates along with those for the mean/mean and mean/sigma methods. The former were obtained by plugging the estimates of the $a_{i}$ and $b_{i}$ parameters into (6) and (9); the latter by plugging the estimates of the $b_{i}$ parameters into (7) and (9). The standard errors for these two methods were calculated from the (co)variances for the item parameter estimates in Table 2 using the same the multivariate delta method. The differences between the results for all three methods were generally substantial, with the precision-weighted method being uniformly best. Especially the results for the *v* parameter are revealing. Whereas the precision-weighted method produced an acceptable low standard error for it, the other two methods lagged behind considerably. In more practical terms, these results suggest that, even with 20 common items, these two methods are likely to seriously misspecify the location of the parameters mapped from one calibration onto the values they would have obtained in another. The extremely large errors for the mean/sigma method are assumed to be due to its ignoring of the unique information in the estimates of the $a_{i}$ parameters.

**Table 4:** Overall estimates of linking parameters and their standard errors.

Method	$\widehat{u}$	$\widehat{\sigma }_{u}$	$\widehat{v}$	$\widehat{\sigma }_{v}$
Precision-weighted average	1.226	0.026	$-0.684$	0.023
Mean/mean	1.237	0.027	$-0.706$	0.078
Mean/sigma	1.197	0.118	$-0.696$	0.084

**Fig. 2:**
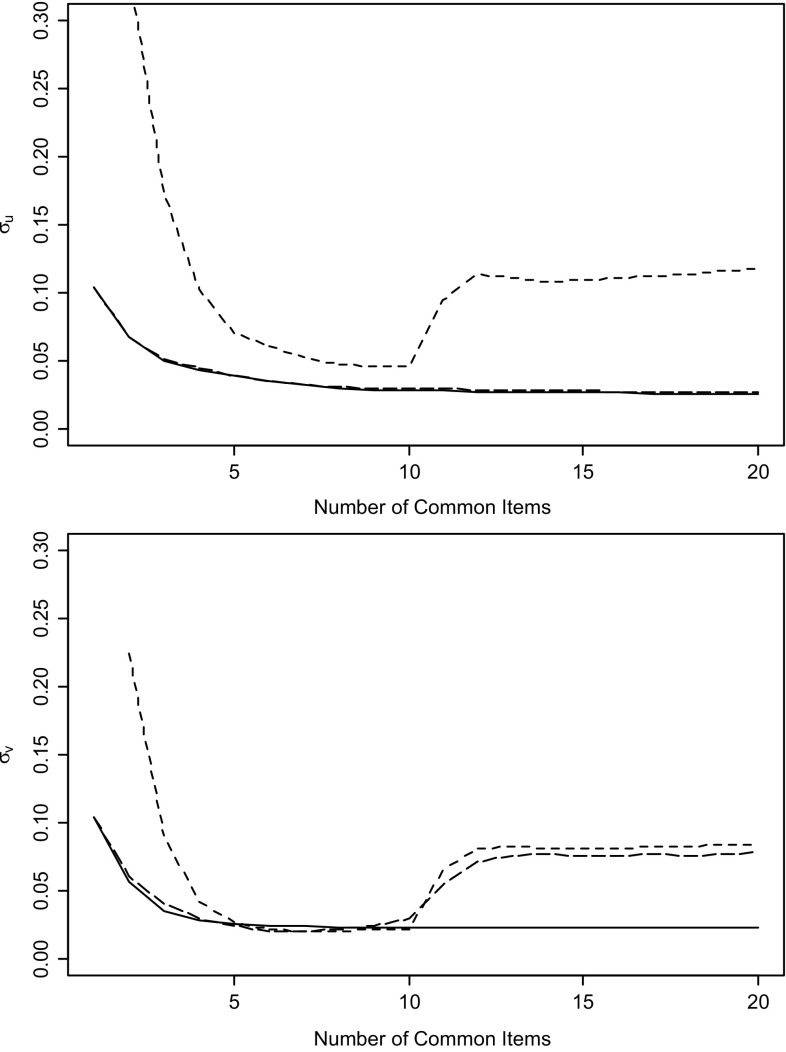
Estimated standard errors for linking parameters *u* and *v* for the precision-weighted (*solid*), mean/mean (*longdash*), and mean/sigma methods (*shortdash*) as a function of the number of common items in the linking design.

It is also interesting to inspect how these overall estimates of the standard errors behave as a function of the number of common items. The curves in Fig. 2 were obtained by adding the common items to the linking design, one at a time beginning with the first item in Table 3. The precision-weighted method produced results that were generally substantially better and never worse than those for the mean/mean and mean-sigma method. In fact, it already reached stability for both estimates after some five common items, whereas there still was considerable room for the other two methods to converge. Also, observe the lack of monotonicity in the curves for the standard errors of *u* and *v* for the mean/sigma method and in the one for the standard error of *v* for the mean/mean method. Whereas one would expect a decrease of them with the extra information in each common item added to the linking design, these methods actually showed a considerable increase for the eleventh and twelfth item. Finally, use of the precision-weighted method with the first ten items in Fig. 2 would yield linking estimates already superior to those for all 20 items for the mean-mean and mean-sigma methods.

**Fig. 3:**
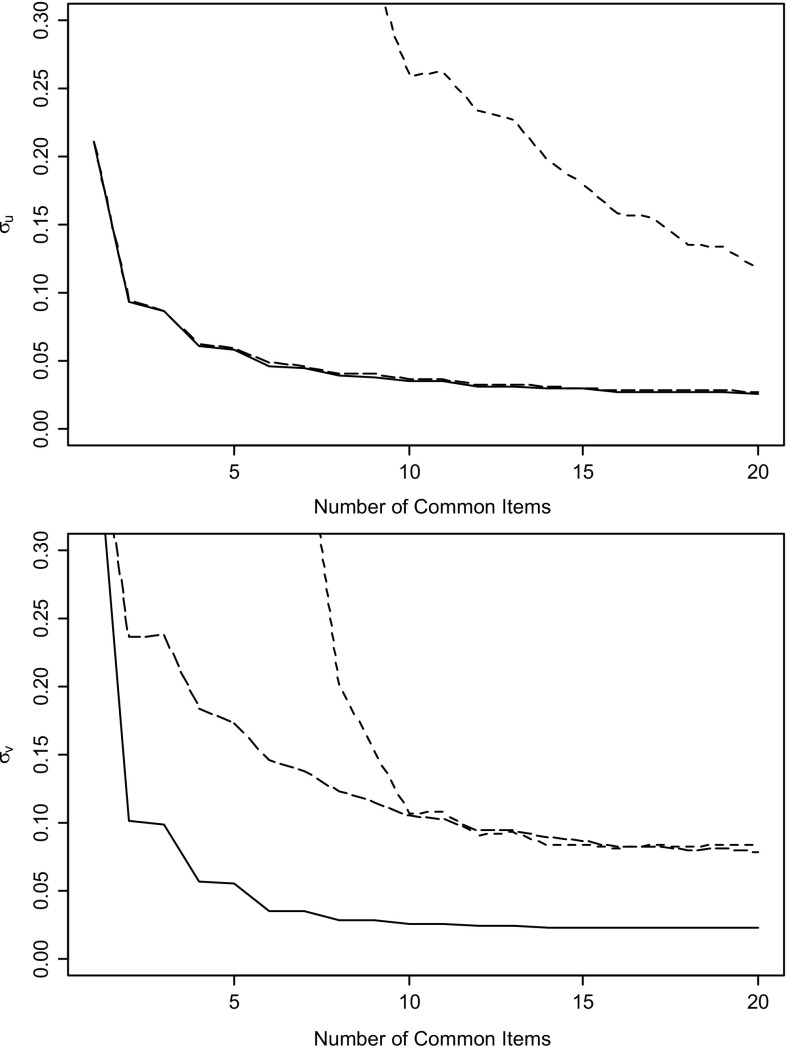
Estimated standard errors for linking parameters *u* and *v* for the precision-weighted (*solid*), mean/mean (*longdash*), and mean/sigma methods (*shortdash*) as a function of the number of common items in the linking design for a different order of the items than in Table 2.

The results in Fig. 2 highlight the importance of the relative precision of the linking parameter estimates contributed by each individual item to the linking design. Surprisingly, if we had added the items to the linking design in a different order, different results would have been found. Figure 3 illustrates the linking errors for the same total set of common items as in Fig. 2, but now added by increasing item difficulty rather than increasing item discrimination as in Table 2. Note that, of course, the overall error associated with all 20 common items remains the same. But the final result is now reached along different trajectories for all three methods. The difference between the results in Figs. 2 and 3 suggests further research on the use of optimal design principles to find the best possible subset of linking items for the linking design from the larger set of candidate items typically available in practical situations.

This example was only to explore what might be possible once the problem of parameter linking in IRT has been provided with a solid formal foundation. Research on the new estimation method, including comparative studies with other linking methods using empirical data, attempts to explain the aberrances produced by the traditional linking methods, and an analysis of the consequences of the confounding of linking error with estimation error in the $c_{i}$ parameters by the response-function methods is currently conducted. Also, as just noted, further research is needed on how to find the optimal linking design given a set of candidate common items.

## Concluding Remarks

We began this paper with a review of the traditional conception of parameter linking in IRT, which appears to have been motivated largely by the notion of equating $\theta$ scores on scales with an indeterminate zero and unit, that is, interval scales in the tradition of Stevens’ (1946) classification. An obvious way to remove the indeterminacy of a $\theta$ scale, according to the tradition, would be to set its zero and unit equal to the mean and standard deviation of the abilities for a population of test takers, and the only reason why we need to link parameters from different calibrations would be to correct for differences between population distributions. Besides, because they are not assumed to be affected by this choice of zero and unit, the $c_{i}$ parameters could be treated as invariant. Lord (1980, sect. 3.5) is quite direct in his claim as to this last point.

The fundamental notion underlying the necessity of parameter linking in IRT, however, is not that of an “interval scale” for the $$\theta $$ parameters in the response model, but possible lack of identifiability of any of its parameters. We have been able to present several results due to this reconceptualization. First, although never formally derived before, the linking functions for the standard reparameterization of the 3PL model (Theorem 4) appear to have the general linear form for the $$a_{i}$$, $$b_{i}$$, and $$\theta _{p}$$ parameters assumed in the current literature, but with the different slope and intercept parameters *u* and *v* in (37) and (38), respectively, and the component function $$\varphi _{c}(c)=c$$ added for the $$ c_{i}$$ parameters. The definitions of *u* and *v* allowed us to derive the different solutions for a few minimal linking designs in (44)–(48). Second, the alternative slope-intercept reparameterization of the 3PL model appears to have a serious impact on the linking problem. Not only have the linking functions for its $$\alpha _{i}$$, $$ \beta _{i}$$, and $$\vartheta _{p}$$ parameters a different form with one more unknown linking parameter *w* (Theorem 6), its parameters are identifiable only for the unpractical type of design that has both common items and common persons (Theorem 7). Third, as a more general result, we now know from Theorem 3 that for any other monotone, continuous response model, the linking functions take the general form of a componentwise monotone vector function—a fact that will simplify our explorations of the linking functions required for nearly every other IRT model currently used in educational and psychological testing. Fourth, although all linking functions were derived for true model parameters and their subsequent estimation was not the focus of this paper, it is already clear that we will have to deviate from the estimation methods currently practised. For instance, as illustrated by our example, rather than estimating linking parameter *u* as the ratio of the mean of estimates of the $$a_{i}$$ parameters in (6) for the common items, it is much more efficient to use an estimate based on the (precision-weighted) mean of their ratios. Fifth, the derivation of the linking function for the $$c_{i}$$ parameters in (35) helps us to evaluate their role in the currently used estimation methods discussed in the introductory section. The Stocking-Lord and Haebara methods admit estimation error in the $$c_{i}$$ parameters into their estimates of the linking parameters, but the mean/mean and mean/sigma methods ignore these parameters entirely. At first sight, the lack of identifiability of the $$c_{i}$$ parameters seems to suggest the choice of a method from the former rather than the latter category. But the fact that their linking function is the identity function $$\varphi _{c}(c)=c$$ implies that, once they have been made identifiable, no further linking is necessary. Consequently, unlike the mean/mean and mean/sigma methods, the Haebara and Stocking-Lord methods confound linking error in the $$a_{i}$$, $$ b_{i}$$, and $$\theta _{p}$$ parameters with estimation error in the $$c_{i}$$ parameters.

Any choice of identifiability restrictions has an element of arbitrariness to it, and the practice of making the 3PL model identifiable using restrictions that include the mean ($\mu _{\theta }=0)$ or standard deviation ($\sigma _{\theta }=1)$ of the ability parameters for the test takers in the calibration study therefore cannot be wrong. Nevertheless, the reliance on the notion of randomly sampling from some population of test takers sometimes automatically associated with it is potentially dangerous. For instance, it easily leads to the idea that we now estimate a population mean and standard deviation and therefore have to account for their sampling error. Indeed, a recent study advocated this idea, along with the claim that large-scale educational assessments tend to overlook the design effects on linking error due to the typical clustering of test takers during sampling (Doorey, 2011, p. 6). However, as demonstrated by Theorem 4, the shape of the true linking functions for the 3PL model does not depend on the actual identifiability restrictions imposed on the calibration studies, let alone on any population parameters adopted for them, or even a specific choice of sampling design used to estimate such parameters.
